# Susceptibility to Oxidation of Selected Freshwater Fish Species Lipids as a Potential Source of Fish Oil in Dietary Supplements

**DOI:** 10.1155/2021/7191639

**Published:** 2021-08-20

**Authors:** Grzegorz Tokarczyk, Grzegorz Bienkiewicz, Patrycja Biernacka

**Affiliations:** West Pomeranian University of Technology, Szczecin, Papieża Pawła VI 3, 71-459 Szczecin, Poland

## Abstract

Polyunsaturated fatty acids from the omega 3 family are more and more often supplied to the diet in the form of supplements. The aim of the study was to characterize the lipid fractions of predatory freshwater fish, i.e., pike (*Esox lucius* L.), perch (*Perca fluviatilis* L.), and pike perch (*Sander lucioperca* L.), and omnivorous fish, i.e., bream (*Abramis brama* L.) and roach (*Rutilus rutilus* L.). Their technological usefulness in terms of the source of fish oil was determined, depending on the rate and degree of their oxidative changes. UV radiation (photooxidation test) was used as a factor accelerating lipid oxidation. Research has shown that selected species of freshwater fish are characterized by high lipid oxidative stability, due to the availability and speed of delivery to the processing plant. The initial level of lipid oxidation of their meat, expressed by the TOTOX index, in any species did not exceed the value of 7, while the acceptable value was 26. The oil obtained from the meat of omnivorous fish after the photooxidation process was characterized by significantly better oxidative stability compared to the oil from the meat of predatory fish. The oxidation resistance of omnivorous fish oil was shown to be higher than that of predatory fish.

## 1. Introduction

Fish are a source of complete proteins and nutritionally valuable fats. They are the primary source of long-chain polyunsaturated fatty acids of the omega 3 family (LC n-3 PUFA). However, for fats to be a valuable source of bioactive ingredients in processed foods, the raw material from which they are produced must be fresh and of high quality [1]. Long-chain omega-3 (n-3) polyunsaturated fatty acids (LC-PUFA), i.e., eicosapentaenoic (EPA, 20: 5n-3) and docosahexaenoic (DHA, 22: 6n-3), are often used as essential components of a healthy, balanced diet, favoring development of the body and alleviating a number of pathological conditions. However, their global supply from all traditional sources of these nutrients is insufficient to meet human nutritional demands.

The type and degree of processing of the fish, as well as the time and method of storage, significantly affect the quality of lipids, especially the degree of their hydrolysis and oxidation [2]. Fish lipids, due to the content of unsaturated fatty acids, are very susceptible to oxidation and to autoxidation processes. The rate of these changes depends, among others, on the species of fish, catch period, or the fatty acid composition [3–5], which may be the cause of completely different changes in the quality of lipids of individual fish species. Fish lipids are more and more often delivered to humans through supplements made of fish oils from various species, which are assigned a number of important functional properties. A very important factor in this regard is the analysis of the degree of fresh and stability of lipids obtained by extraction from tissues and used in dietary supplements or directly for food fortification [6]. The course of oxidative changes in fish lipids in the tissue may be completely different from their changes in the oil, which may be, e.g., dietary supplement. Fish lipids in the muscle tissue, in the processes of oxidation and hydrolysis, can undergo completely different changes than in the oil, where the main factor accelerating and catalysing oxidation is UV radiation. In the tissue, however, many other pathways of transformation may take place, resulting, e.g., from the catalysing effect of haemoglobin [7], the interaction of oxidation products with other food components [8], or the use of additional antioxidant ingredients, such as yeast (*Saccharomyces uvarum*) [9].

Fish oil is mainly obtained from whole fish or liver. Fish by-products (especially from fatty fish) are also increasingly used, but due to the quality of the oil obtained from such raw material, it may not always be suitable for human consumption [10, 11]. In recent years, salmon has become the most popular raw material from which fish oil is obtained, especially production waste left over from salmon filleting, classified in the first category. However, the poor quality of such raw material has a significant impact on the quality parameters of the obtained oil, which often disqualifies it as an ingredient of a dietary supplement. Mason and Sherratt [12] studied the quality of the most popular dietary supplements, rich in omega 3 acids, in the US market. They showed generally that available fish oil-based omega 3 acid supplements were highly oxidized compared to prescription preparations. Primary, secondary, and total oxidation products of lipids in the US market products exceeded the maximum levels established by international quality standards in fish oils, but not in prescription omega 3 preparations. These data indicate that fat oxidation levels found in typical dietary supplements may interfere with their intended effects.

According to Fish Information and Services, freshwater fish catches excluding aquaculture are estimated at 12 million tons live weight in 2018, and in Poland, it was about 58 thousand tons [13]. Such raw material is often characterized by low technological usefulness due to high species diversity, difficulties in mechanized processing, and poor slaughter yield. However, it is a raw material of high fresh, often supplied even in the form of fresh fish, without the need to freeze it. Keeping in mind the economic and technological aspects, it was assumed that freshwater fish, especially the less economically valuable ones can be an excellent raw material for the production of high quality fish oil, which can be used as a dietary supplement or directly intended for food fortification.

In this study, it was assumed that the lipids of predatory and omnivorous fish from the same reservoir will differ in the composition and quality of lipids, which may transfer into the shelf life and quality of lipids, which may transfer into the shelf life and quality of their fat as a the potential source for dietary supplements.

The aim of this study was to characterize the lipid fraction of selected freshwater fish species and to determine their technological usefulness, determined by the rate and degree of lipid oxidative changes. In this study, UV radiations were used as a factor accelerating lipid oxidation (photooxidation test).

## 2. Materials and Methods

The study material consisted of 5 freshwater fish species. These were predatory species, pike (*Esox lucius* L.), perch (*Perca fluviatilis* L.), and pike-perch (*Sander lucioperca* L.), and omnivorous fish, bream (*Abramis brama* L.) and roach (*Rutilus rutilus* L.). Fish were caught in the Szczecin Lagoon between April and October 2019. The fish were delivered to the laboratory packed in ice in the *rigor mortis* condition. After discarding mechanically damaged individuals, fish were pretreated into skinless fillets. Twenty fish of each species were intended for analysis. The fillets were minced on a HENDI 350 meat grinder with a mesh diameter of 5 mm. Laboratory tests were carried out on the samples prepared in this way.

Lipids were extracted using the method of Bligh and Dyer [14], and the fat content was determined by the method of Smedes [15]. Lipids extracted from individual fish species were subjected to a photooxidation test by dosing into Petri dishes with a diameter of 5 cm, such an amount of extract to obtain a layer of approx. 0.5 g of lipids after evaporating the solvent. The samples prepared in this way were exposed to UV light (*λ* = 320 nm) for 30, 60, 90, and 120 min using a CAMAG_30003 UV lamp. The nonirradiated lipids were the reference samples. In all samples, peroxide value (PV) according to Pietrzyk [16], anisidine value (AV), and the TOTOX index = 0.26 · LN + LA according to ISO 6885: 1988 [17] were determined. Moreover, fatty acid composition in initial samples and after 120 min of photooxidation was determined by gas chromatography (GC), using gas chromatograph Agillent Technology 7890A, USA, coupled with mass detector.

Fatty acid methyl esters (FAME) were obtained from the tissue by alkaline hydrolysis of extract of lipids with 0.5 N sodium methylate (CH_3_ONa) according to AOAC [18] and then separated in a 100 m long capillary column coated with Supelco's SP2360 stationary phase with an internal diameter of 0.25 *μ*m. The carrier gas was helium, flowing at a rate of 1 cm^3^/min, the temperature of the injector and detector was 220°C, and the total analysis time was 45 min [19]. Identification of individual fatty acids was based on the comparison of retention times and mass spectra of the particular FAMEs of the sample with those of analogous FAME standards by Sigma company (Lipid Standard). As an internal standard, C 19 : 0 was used. Additionally, water content by gravimetric method, drying the sample at 105°C [20] and the protein content by the Kjeldahl method [21] were determined.

The results presented in the tables and figures are the mean values of triplicate analyses. Statistical analysis was performed using the Statistica 13.0 program. The significance of the differences between the mean values was verified by Duncan's test at the significance level of *p* = 0.05.

## 3. Results and Discussion

### 3.1. Characteristics of the Raw Fish

The fish for research were delivered to the laboratory in the *rigor mortis* condition, which guaranteed the highest freshness and was reflected in the quality assessment. Organoleptic evaluation showed no quality defects, and therefore, the TVB-N (Total Volatile Basic Nitrogen) was not determined. The fish were characterized only in terms of the content of fat and water protein (Table 1), i.e., parameters important for the evaluation of the technological suitability of the raw material as a potential source of fish oil used in the production of dietary supplements or food supplementation. The most significant differences in the content of these components were related to fat, i.e., predatory fish were characterized by much lower fat in meat than omnivorous fish. In the meat of predatory fish, fat constituted from 1.41 to 1.87%, while in the meat of roach and bream are 3.92 and 4.84%, respectively. The differences in the fat content between these species are mainly related to their way of living [22, 23].

### 3.2. Characteristics of the Dynamics of the Oxidation Process Catalyzed by UV Radiation

UV radiation as a catalyzing factor in research on the rate and dynamics of lipid oxidation is a test that can be used in rapid analyses characterizing changes in lipid oxidation. There is a correlation between the time of photooxidation and the storage time of lipids. Research in this area was conducted, i.a., by Bienkiewicz et al. [24]. They investigated the effect of UV radiation time on quality changes of linseed oil in room conditions, packed in films with different levels of UV radiation barrier. In this experiment, changes in the TOTOX index and the loss of ALA (alpha linolenic acid) were assessed. The authors showed that these parameters are a good indicator for the qualitative assessment of fat subjected to accelerated oxidation processes, e.g., through photooxidation with UV radiation.

**Figure 1:**
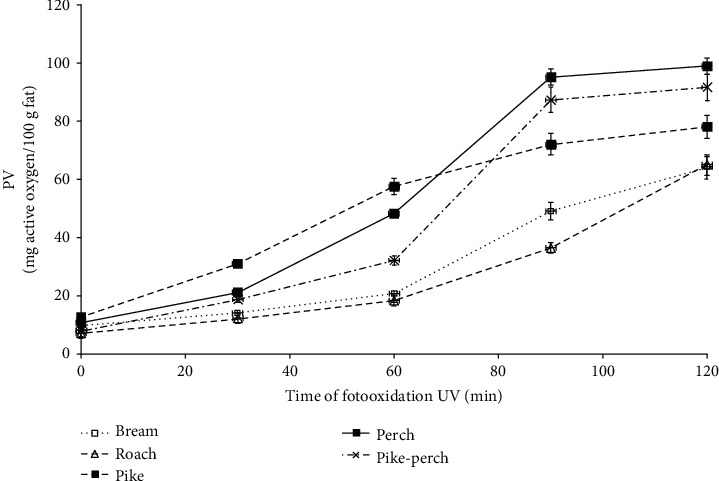
The dynamics of peroxide value (PV) changes during lipid photooxidation of studied fish species.

The dynamics of primary oxidation products of fish lipid changes catalyzed by UV radiation, expressed as a peroxide value (PV), is presented in Figure 1. The initial level of lipid oxidation of the studied species was very similar and amounted to 7.25 mg active oxygen per 100 g fat for roach to 12.65 mg active oxygen per 100 g for pike. As a result of photooxidation, the amount of peroxides increased steadily to 30 minutes for all tested fish species, initiating the autoxidation process. After this time, the process of dynamic oxidation begins and a cascade of reactions with each new active radical molecule increases, increasing the rate and variability of the reaction [25]. Only between 30 and 90 minutes of photooxidation a more dynamic increase in the level of peroxides was observed for predatory fish and slightly smaller for roach and bream. Further exposure time slowed down the dynamics of fat oxidation in predatory fish, while in the fat of roach and bream, an intensive increase in the amount of peroxides was still observed, reaching the level of about 65 mg active oxygen per 100 g lipids, while in predatory fish, this level was from 78 mg active oxygen per 100 g in pike to over 90 mg of active oxygen per 100 g for perch and pikeperch. Such dynamics and differences in oxidation rate between the compared species result from their biological characteristics (predatory and omnivorous fish). Moreover, it may also result from different presence of factors catalyzing oxidative processes in extracted lipids. The amount of prooxidative substances in lipids depends on the presence of such components as iron ions, myoglobin, and oxidation products of myoglobin coming mainly from dark muscles, which are more in predatory fish than in omnivores. In fish, myoglobin oxidation and lipid oxidation are related and interact, and deoxyHb is a stronger catalyst for lipid oxidation compared to oxyHb [26–28]. The formation of deoxy Hb releases iron from inside the porphyrin group, which is a catalyst for lipid oxidation and formation of primary oxidation products, including those catalyzed by UV radiation and lipid-protein interactions [29].

**Figure 2:**
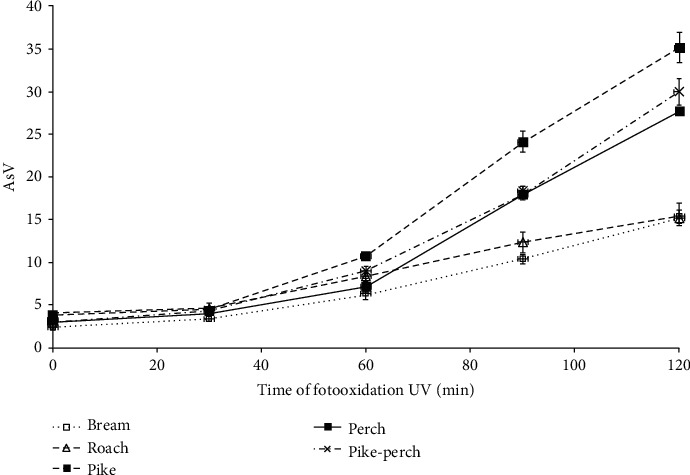
The dynamics of anisidine value (AsV) changes during lipid photooxidation of studied fish species.

Changes in secondary oxidation products expressed as anisidine value (AsV) were similar to changes in the primary oxidation products (Figure 2). In this case, however, the increase of AsV proceeded with the same intensity up to 60 min for all species, while from 60 to 120 min, more secondary oxidation products were formed in the fat of predatory fish than in the fat of roach or bream. The continuous increase in the amount of carbonyl compounds resulted from the fact that in the model study, lipids extracted from the tissue were subjected to photooxidation, so the reactive secondary oxidation products could not interact, e.g., with proteins, as is the case in tissue. In the carbonyl amine reaction, aldehydes can bind directly to amino groups in proteins through covalent bonds which lowers the functional properties of proteins [30]. In this case, there was no inhibition of AsV growth dynamics for the fat of predatory fish, as it was observed in the case of PV.

To visualize the full level of oxidation occurring during the photooxidation process, the total lipid oxidation index (TOTOX) was determined (Table 2). At the beginning of the process, there were no significant differences in the initial lipid oxidation levels of the studied species, only from the 60th minute onwards significant differences between predators and omnivorous fish were observed. Until this time, oil oxidation levels did not exceed the Codex Alimentarius TOTOX limits of ≤26 [31], and for roach and bream, this level did not changed even until the 90th minute of photooxidation (Table 2). These differences, with some fluctuations, were observed until the end of the exposure to UV irradiation time.

Table 3 shows the equations of the total oxidation trend line for the photooxidation process. It was found that all dependencies can be described by linear equation (*y* = *ax* + *b*) with a very high correlation coefficient *R*^2^. When analyzing the equations, and especially the “*a*” parameter, it can be observed that the dynamics of total oxidation level changes for predatory fish was almost twice as high as for roach and bream.

### 3.3. Analysis of Fatty Acid Composition

Lipid oxidation is a key factor leading to the deterioration of food quality, mainly those foods that contain large amounts of unsaturated fatty acids [32]. Analyzing the fatty acid composition of the lipids of the analyzed fish species, the highest proportions of n-3 fatty acids in the lipids of perch, pike, and pikeperch were found. Slightly less proportion of n-3 fatty acids was found in the lipids of roach and the lowest proportion in the lipids of bream (Table 4). However, taking into account the fat content, which is more than twice as high in bream and roach compared to the other species, the amount of n-3 acids extracted from the lipids of these species is comparable. Fish species representing lower trophic levels, especially plant feeding/omnivorous fish such as carp, roach, and bream, obtain n-3 fatty acids mainly from food of plant origin. This is because they have a better ability to digest cell walls, due to the presence of cellulose-digesting enzymes in the gut of endogenous, or more likely, microbial origin [33]. This type of food will determine the composition of fat, as well as its resistance to oxidizing factors. The content of fatty acids, especially of the n-3 family, also depends on environmental and biological factors. As demonstrated in the research by Pedro et al. [23], the amount of EPA + DHA was related to lipid content, but was more dependent for species with higher trophic positions, e.g., pike and perch (average 37% contribution to total fatty acids), than for species with lower trophic positions, such as roach or bream presented in the paper (average about 15% contribution to total acids). Another issue affecting fatty acid composition and susceptibility to lipid oxidation arises in the analysis of aquaculture fish species. An example of such a fish would be salmon, especially in the form of meat obtained after filleting the fish, which is commonly used for oil production. As shown by Sprague et al. [34] and De Roos et al. [35], the limited availability of feed rich in LC n-3 PUFAs and their replacement by feed with plant additives resulted in a significant reduction of EPA and DHA acids in salmon oil in the last years. Replacing n-3 PUFAs with n-6 PUFAs in the diet of salmon and using their oil for dietary supplements result in deepening the incorrect ratio of n-6 to n-3 fatty acids in humans. In the presented results (Table 4), this ratio is very favorable for all species tested, especially considering the continuous deficiency of LC n-3 fatty acids in the human diet.

The percentage share of these acids in reputable dietary supplements sold in the USA range from 30% to 70% [12] and in the preparations available on the Polish market at the average level of about 40%. However, it should be remembered that these are oils that undergo many refining processes, which causes, among others, an increase in the share of n-3 acids, often at the cost of losing their oxidative stability.

As a result of the UV-catalyzed photooxidation process, their amount is reduced by an average of five percentage points for predatory fish and by less than 2 percentage points for roach and bream. Such a dependence may indicate that species of omnivorous fish, also classified as economically low valuable, may be a good raw material for obtaining oil for the purposes of dietary supplementation. The most important factors that may influence lipid rancidity in fish muscles are high PUFA content and the presence of prooxidants, especially those containing haem groups [36]. In the case of the compared species of fish, it can be explained by the difference in the amount of light and dark muscle, which is the main source of haem compounds getting into the oil during extraction.

## 4. Conclusions

Wild freshwater fish are eaten less frequently than saltwater and aquaculture fish such as salmon and trout. It is caused, i.a., by more limited availability, the volume of supply, and most of all difficulties in pretreatment by limiting the possibility of automating processes and culinary processing due to the large number of pin bones, large and hard rib bones, fish scales, etc. Fish species classified as economically low valuable, such as roach or bream, are not often used. In this study, it was shown that freshwater fish species obtained from local waters are characterized by high nutritional value and good quality and freshness. Moreover, the initial lipid peroxidation level of their meat, expressed as TOTOX, did not exceed 7 in any species, with an acceptable value of 26. These fish are also a valuable source of protein, with an amount of 17% to over 20%. The amount of n-3 fatty acids, taking into account the fat content in meat, ranges from 0.35 g per 100 g of tissue for roach to about 0.55 g per 100 g of tissue for perch or pikeperch. While predatory fish species with low calorific value, characterized by very low fat content but rich in n-3 fatty acids, are more eagerly used for culinary purposes, species such as roach or bream, apart from marginal industrial use, can be valuable raw material for obtaining fish oil, as a source of n-3 acids. Moreover, this oil is characterized by a high degree of fresh and much better oxidative stability compared to oil from meat of predatory fish.

## Figures and Tables

**Table 1:** Protein, fat, and water content in the flesh of the tested fish species.

Species	Protein (%)	Water (%)	Fat (%)
Bream	17.71 ± 0.11	74.67 ± 0.30	4.84 ± 0.05
Roach	20.53 ± 0.14	72.86 ± 0.37	3.92 ± 0.23
Pike	17.80 ± 0.59	75.81 ± 0.54	1.41 ± 0.22
Perch	18.93 ± 0.25	77.52 ± 0.24	1.53 ± 0.12
Pike-perch	19.77 ± 0.33	75.06 ± 0.44	1.87 ± 0.21

**Table 2:** Total oxidation of fat values (TOTOX) for the studied fish species depending on the time of photooxidation.

Species	Time of oxidation (min)				
	0	30	60	90	120
Bream	4.83 ± 0.12^a^	7.13 ± 0.21^a^	11.73 ± 1.12^a^	23.18 ± 1.47^a^	32.01 ± 2.05^a^
Roach	6.00 ± 0.14^a^	7.96 ± 0.23^a^	13.15 ± 0.63^a^	21.81 ± 2.04^a^	32.34 ± 3.22^a^
Pike	7.14 ± 0.21^a^	12.43 ± 0.47	25.70 ± 1.32	42.82 ± 3.12^b^	55.40 ± 2.89^b^
Perch	5.84 ± 0.21^b^	9.46 ± 0.14^b^	19.67 ± 0.57^b^	42.62 ± 1.33^b^	53.37 ± 3.45^b^
Pike-perch	5.27 ± 0.19^a^	9.39 ± 0.24^b^	17.40 ± 0.70^b^	40.78 ± 3.08^b^	53.80 ± 3.41^b^

^a, b, c, d^No significant differences in total oxidation level (TOTOX), between species and for the same photooxidation time.

**Table 3:** Comparison of the equations characterizing process of the dynamics of total oxidation (TOTOX) catalyzed by UV radiation for the tested fish species.

Species	Equation	Correlation coefficient *R*^2^
Bream	*y* = 0.2347*x* + 1.6917	0.936
Roach	*y* = 0.2218*x* + 2.9449	0.935
Pike	*y* = 0.423*x* + 3.3176	0.975
Perch	*y* = 0.4275*x* + 0.5441	0.942
Pike-perch	*y* = 0.4281*x*–0.3584	0.932

**Table 4:** Percentage share of individual groups of fatty acids before and after photooxidation.

Group of FA	Bream		Roach		Pike		Perch		Pike-perch	
	Time of photooxidation (min)									
	0	120	0	120	0	120	0	120	0	120
SFA	27.22	28.45	30.12	33.02	30.52	31.96	29.34	29.75	35.98	37.45
MUFA	57.87	59.12	39.23	41.58	29.28	32.14	22.15	25.08	25.47	29.34
PUFA	14.92	12.43	30.65	25.40	40.20	35.90	48.51	45.17	38.55	33.21
∑n-3	9.06^aA^	7.76^aB^	22.02^cA^	21.16^cA^	37.11^bA^	31.15^bB^	37.65^bA^	32.71^bB^	34.12^dA^	28.44^dB^
∑n-6	5.85^aA^	4.68^aB^	8.63^cA^	5.34^cB^	3.09^eA^	4.75^eB^	10.86^bA^	12.46^bB^	4.38^dA^	4.77^dA^
n6/n3	0.65	0.60	0.39	0.26	0.08	0.15	0.29	0.38	0.13	0.17

^∗^Relative error did not exceed 5% between repetitions *n* = 3. ^a, b, c, d, e^Significant differences for the sum of n-3 and n-6 acids, between species and for the same photooxidation time. ^A, B^Significant differences for the sum of n-3 and n-6 acids, within species and for the different photooxidation time.

## Data Availability

The data used to support the findings of this study are available from the corresponding author upon request.
